# The liver compensates for zonal loss of glucose 6-phosphatase to prevent glycogen storage disease

**DOI:** 10.64898/2026.07.26.740786

**Published:** 2026-07-28

**Authors:** Roger Liang, Ling Cai, Phong T. Nguyen, Sherwin Kelekar, Maggie B. Cervantes, Trevor Tippetts, Eric Chen, Roberto Ribas, Alison Ryan, Janice Chou, Ramon Sun, Hao Zhu, Ralph J. DeBerardinis

## Abstract

The liver is organized into spatial zones with distinct metabolic roles, but how this architecture contributes to disease remains unclear. Glucose production is concentrated in periportal hepatocytes and depends on G6PC1; loss of this enzyme causes glycogen storage disease type Ia (GSD1a). We tested whether G6PC1 loss in specific zones, including its primary periportal location, is sufficient to cause disease. Unexpectedly, loss of *G6pc1* in any single zone caused local glycogen accumulation but did not produce the systemic metabolic abnormalities or liver tumors seen after whole-liver deletion. Instead, disease developed only after near-complete loss of *G6pc1* across the entire liver. These findings show that organ-wide compensation preserves metabolic homeostasis and suppresses tumorigenesis despite localized disruption.

**Teaser:**

Spatial *G6pc1* loss causes local glycogen storage without systemic metabolic disease.

## Full Text Availability

The license terms selected by the author(s) for this preprint version do not permit archiving in PMC. The full text is available from the preprint server.

